# Factors causing timely referral for fetal echocardiography in the final diagnosis of congenital heart malformations: A cross-sectional study

**DOI:** 10.18502/ijrm.v20i6.11443

**Published:** 2022-07-06

**Authors:** Mojgan Barati, Nahal Nasehi, Sareh Aberoumand, Mahin Najafian, Abdolrahman Emami Moghadam

**Affiliations:** ^1^Fertility, Infertility, and Perinatology Research Center, Ahvaz Jundishapur University of Medical Sciences, Ahvaz, Iran.; ^2^Department of Pediatric Cardiology, Golestan Hospital, Ahvaz Jundishapur University of Medical Sciences, Ahvaz, Iran.

**Keywords:** Echocardiography, Fertilization, Heart diseases, Pregnant women.

## Abstract

**Background:**

Congenital heart disease (CHD) is one of the most frequently inherited illnesses associated with adverse outcomes.

**Objective:**

This study aimed to determine the referral cause for fetal echocardiography in the final diagnosis of major CHD.

**Materials and Methods:**

In this cross-sectional study, the data of 1772 pregnant women, referred to a diagnostic clinic during 2017-2020, were reviewed. Data were collected from participants on maternal age, gestational age, history of previous child's heart disease, body mass, the order of birth of children (baby birth rank), type of woman's disease, history of poor midwifery, and nuchal translucency (NT).

**Results:**

Of the 1772 pregnant women, only 33 women (1.8%) had a fetus with CHD major. Abnormality in ultrasound (57.6%), history of abortion (36.4%), increased NT and gestational diabetes (18.2%) and gestational diabetes (18.2%) were identified as the most common referral reasons for fetal echocardiography in these women. Other reasons included a previous child with Down syndrome (12.1%), a previous child with heart disease (12.1%), a history of stillbirth (12.1%), hypothyroidism (12.1%), taking medication during the pregnancy period (9.0%), no underlying disease (9.0%), multiple pregnancies (6.0%), diagnosis with high-risk fetal heart disease (3.0%), high-risk combined aneuploidy screening test in the first trimester (3.0%), in vitro fertilizationpregnancy (3.0%), and having a child with an intellectual disability (3.0%).

**Conclusion:**

According to the results, it can be concluded that ultrasound abnormality, abortion, increased NT and gestational diabetes are the most important factors for referring pregnant women for fetal echocardiography.

## 1. Introduction

Congenital heart disease (CHD) is one of the most common cardiac complications during pregnancy and a frequent type of birth defect that is associated with adverse outcomes. This flaw is a type of prenatal cardiovascular illness that includes structural disorders in the heart or large veins. The prevalence of this malformation is about 8 individuals per 1000 living childbirths. Besides, the prevalence of this congenital defect in aborted fetuses, stillbirths and premature infants has been reported as 10-25%, 3-4% and 2%, respectively (1, 2). The prevalence of CHD is 6 and 4 times higher than chromosomal abnormalities and neural tube defects, respectively (3). About 20-30% of cases of CHD are life-threatening and require surgery in the first year of life (4, 5). More than 50% of child mortality attributable to birth defects is related to CHD (6, 7).

Fetal echocardiography is an imaging procedure that provides quality images and detailed anatomy, and is used for the diagnosis of fetal cardiac anomalies (8). The development of fetal echocardiography has made the diagnosis of CHD possible, and evidence suggests that prenatal diagnosis has significantly altered the management of the disease (9, 10). Early detection of heart disorders has many advantages. Prenatal screening and diagnosis increase survival rates and reduce complications (11, 12). Prenatal diagnosis not only identifies heart disorders, but also detects the associated abnormalities; it provides an accurate view of the condition and, if necessary, the possibility of termination of pregnancy (12, 13). However, despite these advances, most prenatal CHD cases remain unidentified, and this is why continuous innovation and advances in technology and training are needed (14, 15).

This study aimed to determine the referral cause for fetal echocardiography in the final diagnosis of major CHD.

## 2. Materials and Methods

### Study setting and participants

The medical records of all pregnant women referred to Narges Clinic, Ahvaz, Iran from January 2017 to January 2020, for fetal echocardiography with any reason (including a history of abortion, ultrasound problems, gestational diabetes, chronic diabetes, history of stillbirth, high body mass, etc.) were reviewed. All files with incomplete information were excluded.

### Variables and measurement

The data included abnormality in ultrasounds, history of abortion, increased nuchal translucency (NT), gestational diabetes, having a previous child with Down syndrome, having a previous child with heart disease, history of stillbirth, hypothyroidism, taking medicine during pregnancy, no underlying disease, multiple pregnancies, high-risk screening, in vitro fertilization (IVF) pregnancy, and having a previous child with an intellectual disability.

### Ethical considerations

This study was approved by the Ethics Committee of Ahvaz Jundishapur University of Medical Sciences, Ahvaz, Iran (Code: IR.AJUMS.REC.1399.392). This study was conducted in accordance with the Helsinki Declaration of 1975, as revised in 2013. The principle of confidentiality of information was fully observed.

### Statistical analysis

In this study, to analyze the information and obtained data, descriptive statistics (frequency and percentage) were examined using the SPSS software version 21.0 (Statistical Package for the Social Sciences, SPSS Inc, Chicago, Illinois, USA).

## 3. Results

Out of 1772 cases reviewed, only 33 women (1.8%) had a fetus diagnosed with major CHD on echocardiography. The mean ± SD of the pregnant women's age and gestational age were 31.7 ± 3.1 yr and 21.4 ± 4.3 wk, respectively. Almost half of the pregnant women with abnormal echocardiography had a normal body mass index (45.5%). Nearly half of all pregnant women were nullipara (48.5%), and more than a third had a history of one-time deliveries (36.4%) (Table I). The main reasons for referring these women for echocardiography were abnormality in ultrasounds (n = 19, 57.6%), and a history of abortion (n = 12; 36.5%), while IVF pregnancies (n = 1; 3.0%), high-risk screening (n = 1; 3.0%) and having a child with an intellectual disability (n = 1; 3.0%) were less frequent cases (Table II).

**Table 1:** Demographic characteristics of participants (n = 33)


**Variables**		**Mean ± SD or number (percentage)**
Maternal age (yr)*		31.7 ± 3.1
Gestational age (wk)*		21.4 ± 4.3
BMI (kg/m^2^)**		
	**Underweight**	3 (9.1)
	**Normal**	15 (45.5)
	**Overweight**	8 (24.3)
	**Obese**	7 (21.2)
Parity**		
	**Nullipara**	16 (48.5)
	**Multipara**	17 (51.5)
**Number of deliveries **		
	**0**	16 (48.5)
	**1**	12 (36.4)
	**2**	3 (9.1)
	**3**	1 (3.0)
	**> 3**	1 (3.0)
*Data presented as Mean ± SD. **Data presented as n (%). BMI: Body mass index		

**Table 2:** Reasons for referral of women with a fetus diagnosed with major echocardiography (n = 33)


**Reason for referral**	**Number (percentage)**
Abnormality in ultrasounds*	19 (57.6)
**History of abortion**	12 (36.5)
**Increased NT**	6 (18.2)
**Gestational diabetes**	6 (18.2)
**Having a previous child with Down syndrome**	4 (12.1)
**Having a previous child with heart disease**	4 (12.1)
**History of stillbirth**	4 (12.1)
**Hypothyroidism**	4 (12.1)
**Taking medicine during pregnancy**	3 (9.0)
**No underlying disease**	3 (9.0)
**Multiple pregnancies**	2 (6.0)
**High-risk screening**	1 (3.0)
**IVF pregnancy**	1 (3.0)
**Having a previous child with intellectual disability**	1 (3.0)
Data presented as n (%). NT: Nuchal translucency, IVF: In vitro fertilization, *Abnormalities included: Single arterial umbilical cord, fetal tachycardia, fetal bradycardia, cerebral choroid plexus	

## 4. Discussion

The aim of this study was to determine the referral indications for fetal echocardiography. In thepresent study, referred pregnant women were 31.7 ± 3.1 yr old, which is similar to other studies (16, 17). But in another study, the mean age of thepopulation was 27.6 ± 4.6, slightlyyounger in age (18).

An important aspect of this study which needs to be highlighted is the gestational age at which the mothers were referred for fetal echocardiography. The mean gestational age at referral in those suspected to have CHD in ultrasonography was 21.4 ± 4.3 wk. This may be late for any decision. Other researchers reported 11-13 wk and on average 20.37 in their surveys, respectively (16, 18). According to another study, the most detected cardiac abnormalities were diagnosed at 16-19 wk of gestation (19). Thus, the timing of referral in our study seems to have been postponed. Detection of a defect or CHD is very important in the early stages of disease progression because in such cases the treatment methods efficiently work (20).

In one study, it was recommended that women with pre-pregnancy diabetes should have an echocardiogram before the 20 ${}^{\text{th}}$ wk for CHD, and that in diabetic women (pregnancy and pre-pregnancy) with low diabetes control, a fetal echocardiogram should be recommended in the third trimester (21). In our study 18.2% of pregnant women had gestational diabetes mellitus.

As mentioned, in this study, it was found that the most common indication for referral for fetalechocardiography in pregnant women referred to the echocardiography department was abnormality in ultrasound (57.6%) and the lowest frequencies were related to having a high-risk screening, IVF pregnancy and having a previous child with an intellectual disability, the frequency of each of which was 1%. The findings of another study support our results (21). Also, in another study, the most common primary indication for referral was abnormal cardiac findings on prenatal ultrasound examinations, which were observed in 54 cases (23.3%) of 276 cases (22).

The next commonest indication for referral in this study was history of abortion (36.5%). Only 18.2% of pregnant women were referred for fetal echocardiography due to increased NT. This may be either because the number of fetuses with increased NT is very low or there is a lack of awareness about the important correlation between increased NT and CHD. NT increases with crown rump length (CRL) and hence is usually measured in percentiles in relation to CRL. The 95 ${}^{\text{th}}$ centile of NT for a CRL of 38 is 2.2 mm and for a CRL of 84 is 2.8 mm, whereas the 99 ${}^{\text{th}}$ centile (3.5 mm) does not change significantly with CRL (23).

Increased NT is well known to be associated with a higher incidence of CHD, and this incidence increases with higher values of NT. One study showed that the CHD prevalence was 0.8/1000 pregnancies when the NT was below the 95 ${}^{\text{th}}$ centile, while it increased to 63.5 per 1000 when the NT was above the 99 ${}^{\text{th}}$ centile (23). Due to the proven correlation between NT and CHD, we would like to impress the need for a uniform pattern of NT measurement and referral for a detailed fetal echocardiogram if the NT value is greater than at least the 99 ${}^{\text{th}}$ centile. Such fetuses should be referred for early fetal echocardiography rather than waiting until 18-20 wk.

Diagnosis of CHD has significant implications for overall fetal health; so where heart ill health is suspected, such as in cases of chromosomal or fetal diseases, fetal non-cardiac abnormalities shown in an ultrasound, fetal heart irregularities, placental coronary placenta, increased NT thickness on an ultrasound, or suspected cardiac abnormalities, it is recommended that a conventional ultrasound and fetal echocardiography are performed, especially as fetal echocardiography does not harm the fetus or the mother (24).

If a fetal echocardiogram shows a problem, since most CHD is treatable, it is necessary to plan for delivery in a hospital that has the necessary facilities for the care of such babies. With the advancement of science, most of the heart diseases observed in the fetus can be prevented or treated, and there is no need to worry (25).

The best time to do fetal echocardiography is before the end of the 18 ${}^{\text{th}}$ wk of pregnancy, because until then if diagnosed with incurable heart disease, it is possible to terminate the pregnancy, but after that, termination of the pregnancy will be unauthorized (26). It is recommended that women get genetic counseling before they become pregnant if they are aware of a specific disease in their family. Proper genetic counseling can prevent the birth defect so that the life of the mother and child will not be affected by a congenital disease (27).

Finally, it is possible for some women to have problems with their fetal heart during pregnancy, some of which include mothers who had a child with a heart problem during a previous pregnancy, those who have a history of CHD, women taking certain drugs (such as medications for blood pressure, diabetes, or lupus), women who became pregnant with assisted reproductive techniques such as IVF, or women with a twin pregnancy. If any problems are seen during the first and second trimesters, a fetal heart echocardiogram should be done to make sure the fetus is healthy.

## 5. Conclusion 

Abnormality in ultrasounds, a history of abortion, and increased NT were identified as the most common reasons for referral for fetal echocardiography. Fetal echocardiography is a non-invasive method for early detection of CHD, which is suitable not only for high-risk pregnancies but also for low-risk pregnancies. Given that in some cases, despite a normal pregnancy ultrasound and lack of indications for echocardiography during pregnancy, we see congenital heart defects after birth, we recommend that fetal echocardiography be routinely performed for all pregnant women.

##  Conflict of Interest

The authors declare that there is no conflict of interest.
